# Scalp acupuncture for stroke: A protocol for an overview of systematic reviews and meta-analysis

**DOI:** 10.1097/MD.0000000000031472

**Published:** 2022-11-04

**Authors:** Sun-Young Park, Hyun-Tae Kim, In Heo, Man-Suk Hwang, Eui-Hyoung Hwang, Byung-Cheul Shin

**Affiliations:** a The 3rd Division of Clinical Medicine, School of Korean Medicine, Pusan National University, Yangsan, Republic of Korea; b Department of Korean Medicine Rehabilitation, Pusan National University Korean Medicine Hospital, Yangsan, Republic of Korea.

**Keywords:** assessment of multiple systematic reviews-2 (AMSTAR-2) tool, overview, randomized controlled trial, scalp acupuncture, stroke, systematic reviews

## Abstract

Scalp acupuncture (SA) is a new acupuncture method that connects head acupoints and aculines, and many systematic reviews (SRs) have been published on its use against neuropsychiatric diseases. However, no overview of SRs on the effectiveness of SA in stroke recovery has been conducted. Therefore, our overview aims to evaluate the methodological bias and reliability of the conclusions of SRs regarding SA for stroke recovery and help clinical decision-makers translate this research into clinical policy and practice.

**Methods:**

We will consider SRs and meta-analyses of randomized controlled trials to evaluate the effects of SA on stroke recovery. Two reviewers will identify relevant studies, extract data information, and assess the methodological quality using the Assessment of Multiple Systematic Reviews-2 tool. The Preferred Reporting Items for Systematic Reviews and Meta-Analyses report checklist will also be included in the study to assess the quality of the reports. We will use evaluations of the Grading of Recommendations Assessment, Development and Evaluation of the authors of the included SRs. The Risk of Bias in Systematic Review tool will be used to assess the risk of bias of SRs. The screening of SRs, eligibility evaluation, data extraction, methodological quality, and quality of evidence will be conducted by independent reviewers in pairs. The outcomes of interest include the Modified Edinburgh–Scandinavian Stroke Scale, Ability of Daily Living, Functional Independence Measure, Barthel index, Fugl–Meyer assessment, clinical effective rate, and adverse events. Data will be extracted using predefined forms designed to summarize the important characteristics of each review. The evidence will be a descriptive synthesis of the type and content of the intervention and the results reported.

**Results:**

The results will be published in a peer-reviewed journal.

**Conclusions:**

We expect to organize evidence from multiple SRs on the effectiveness of SA for stroke recovery and synthesize the findings in an accessible and useful documentation.

## 1. Introduction

The World Health Organization defines stroke as a sudden onset of focal neurological or general disability of vascular origin, lasting more than 24 hours or leading to death.^[1]^ Stroke is largely divided into 3 categories: cerebral infarction due to sudden blockage of cerebral artery, cerebral hemorrhage due to rupture of cerebral artery, and subarachnoid hemorrhage due to cerebral artery hemorrhage in the space between the pia mater and arachnoid.^[1]^

Stroke affects 17 million people worldwide each year, of which 6.5 million die and 5 million become permanently disabled.^[2]^ Stroke, with its high incidence and disability rates, has become the second most common cause of disability worldwide. Stroke is 1 of the 3 primary causes of death in Korea and is a key cause of death in China, as well as in Western countries.^[2]^

The treatment methods for stroke recovery include surgery for hemorrhage, drugs and rehabilitation exercise therapy for infarction. Acupuncture is also used to assist in recovery, however, the evidence on its effectiveness is inconclusive. Unlike general acupuncture, scalp acupuncture (SA) is a new treatment for neuropsychiatric diseases.^[3]^

SA is defined as a modern acupuncture technique that penetrates specific areas of the scalp or lines on the scalp based on a specific method, and not only by applying traditional head acupoints. SA differs considerably from traditional acupuncture in that it has its own theoretical basis and its acupoints are quite different from those of classic acupoints. It contributes to brain activation, motor function improvement, sedation, pain relief, and mental and physical care.^[4]^

In the 21st century, the number of patients complaining of degenerative brain diseases, such as stroke, dementia^[5,6]^ and Parkinson’s disease^[7,8]^ is increasing significantly; therefore, the use of SA therapy among various acupuncture methods can be expected to increase adoption among clinicians if the evidence supports its effectiveness.

Several randomized controlled trials have been performed to investigate the effectiveness of SA treatment on stroke recovery, and multiple meta-analyses based on randomized controlled trials have been conducted. Many meta-analyses have shown that SA treatment has several benefits in patients with stroke.^[9–14]^ However, each systematic review (SRs) does not provide a clear, singular recommendation because of inconsistent results, and no critically designed overview to evaluate SRs of SA treatment on stroke recovery has been carried out to date. Therefore, this review protocol aims to summarize the clinical evidence for SA treatment, and to analyze the methodology and reporting quality of existing SRs in stroke recovery in order to provide clinical researchers, doctors, and patients with information on the credibility of the current evidence, the synthesis of its findings, and directions for future research.

## 2. Methods

### 2.1. Study registration

This protocol follows the recommendations of the Cochrane Handbook for Systematic Reviews of Interventions.^[15]^ This protocol was recorded in the Prospective International Registry of Systematic Review (PROSPERO), registration number CRD42022309463 (https://www.crd.york.ac.uk/prospero/display_record.php?ID=CRD42022309463), prior to conducting an overview of SRs.

### 2.2. Inclusion and exclusion criteria

#### 2.2.1. Types of study.

We will include SRs: reviews that use a systematic search strategy to find relevant research aimed at answering a clearly defined clinical question. We will exclude individual studies, case reports, case series, editorials, and clinical guideline publications.

#### 2.2.2. Type of participants.

Patients of any age, sex, illness duration, or stroke stage will be eligible. They will include cases with SA administered to patients with stroke diagnosed using magnetic resonance imaging or computed tomography or according to the World Health Organization guidelines.

#### 2.2.3. Type of interventions.

Treatment with invasive SA will be used exclusively as an intervention measure. We will exclude the use of classic acupuncture treatments in the head area because its method of treatment does not align with the modern SA theory.

#### 2.2.4. Type of comparator (S)/control.

No sham treatment (i.e., placebo) SA or related conventional therapies will be included. We will include studies in which co-interventions, in addition to SA, were administered equally in both groups.

#### 2.2.5. Types of outcome measurements.

##### 2.2.5.1. Primary outcomes.

Primary outcomes of the measurement will include: Acute/subacute stroke, based on the neurological deficit measure (Modified Edinburgh Scandinavian Stroke Scale); and Chronic stroke, based on measures of functional recovery (Ability of Daily Living, Functional Independence Measure, Barthel index, or modified BI), and Motor function (Fugl–Meyer assessment) etc.

##### 2.2.5.2. Secondary outcomes.

Secondary outcomes will include the clinical effective rate, adverse events, and other relevant clinical variables (such as mortality or hospitalization).

### 2.3. Search methods for identification of studies

We will search any articles containing SRs on the effectiveness of SA for stroke recovery in MEDLINE, Embase, PubMed, Cochrane Library, and Web of Science; in addition, we will also search through 4 databases based in China (China Science Journal Database, China Biomedical Literature Database, the China National Knowledge Infrastructure, and Wan-Fang Database), Korean Medical Databases, and the Japanese Medical Database. All databases will be searched from their inception to the present date. Due to the language limitations of the researchers, we will limit the language of the search literature to English.

### 2.4. Data extraction and management

**Figure 1.**
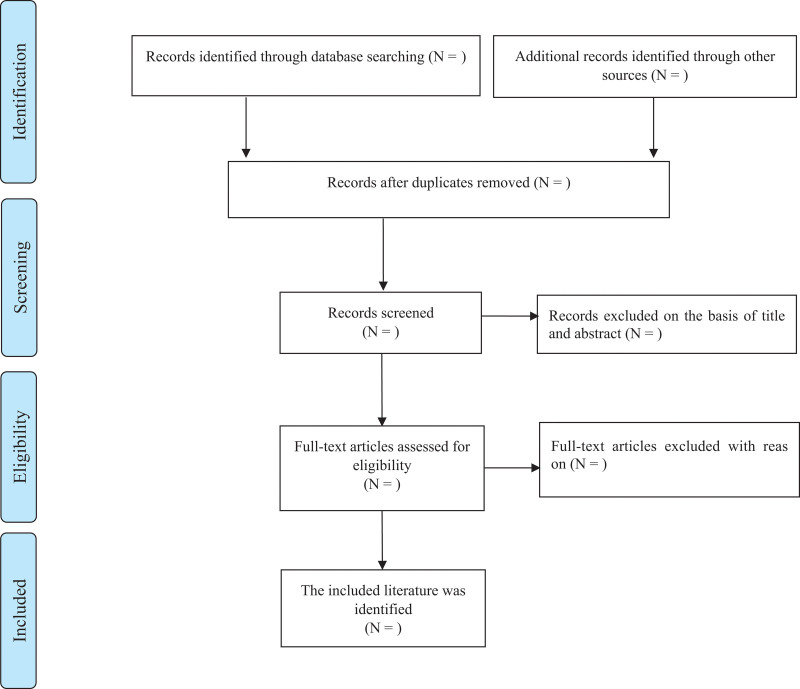
Flow diagram of the study selection process.

The bibliographies obtained from the SRs will be imported into Endnote X8 for management. First, after any deduplication, we will exclude studies that do not meet the inclusion criteria based on screening of the abstracts and titles. Second, 2 reviewers have been selected to extract the data independently. In case of any disagreement, the 2 evaluators will discuss and reach an agreement. The extracted contents from each SR will include data such as the first author, year, intervention measures, control measures, outcome indicators, and risk of bias-related items, which will be recorded in Excel as predefined criteria. The planned selection process is described in a flow chart (Fig. 1).

### 2.5. Risk of bias

Two reviewers will independently appraise the risk of bias of the included SRs using the validated measurement tool in the Assessment of Multiple Systematic Reviews-2 tool checklist by scoring each SR with a maximum of 11 points. Disagreements will be resolved by discussion and/or consultation with a third reviewer.^[16]^ Additionally, Preferred Reporting Items for Systematic Reviews and Meta-Analyses (PRISMA) will be applied to assess the report quality of SRs and MAs. Two authors will evaluate the quality of each study using the PRISMA, a 27-item list. Each checklist item will be evaluated as yes, no, or partially yes to indicate compliance.^[17]^ The evaluation of the evidence quality of the included studies will be conducted by 2 reviewers, using the Grading of Recommendations Assessment, Development and Evaluation (GRADE) approach. GRADE specifies 4 categories: high, moderate, low, and very low.^[18]^ Two authors of this review will assess the risk of bias of the included studies, using the Risk of Bias in Systematic Review (ROBIS) tool. The ROBIS is a tool used to assess the risk of bias of SRs, which involves the assessment of 4 domains: study eligibility criteria, identification and selection of studies, data collection and study appraisal, and synthesis and findings. The evaluation of the risk of bias that is associated with each domain, which will be judged as “low risk,” “high risk,” or “unclear risk.”^[19]^

### 2.6. Strategy for data synthesis

We will incorporate a narrative synthesis of the results of the review, structured around the type and content of the interventions and the reported results. Assessment of multiple systematic reviews-2 will be used for each SR’s methodological quality assessment, PRISMA will be applied to assess report quality, GRADE will be used to assess the quality of evidence, and ROBIS will be used for an assessment of potential bias. The synthesis of these analyses will be conducted in tabular form for each review.

### 2.7. Subgroup analysis

Subgroup analyses will be performed in cases of significant heterogeneity during clinical research. The subgroup criteria may include variables such as the stage of stroke (acute/subacute/chronic), interventions, and control group intervention.

## 3. Discussion

This study provides a protocol for an overview of SRs regarding the effectiveness of SA treatment for stroke recovery. It will summarize the evidence of SA for stroke recovery from a variety of relevant SRs. To the best of our knowledge, this will be the first study in this field to attempt to aggregate evidence of SA for stroke recovery. In the discussion section of the full report of our study, we plan to contain the following subsections, which are typical for this type of study: summary of main findings, strength and limitations, comparison with other studies and opinions, interpretation of results, and conclusions. A potential limitation of the protocol itself is that, if after review of available SRs is complete and there is a scarcity of evidence that can be considered reliable, valid, and unbiased, it will limit the utility of the findings from the final report. Although, if this is the case, it will highlight the need for more thorough and high-quality research to be performed on SA and stroke recovery before recommendations can be made for clinical use. The results of this overview will offer researchers with information about the reliability of the current combined evidence and research directions in the future.

## Author contributions

S-Y Park, B-C Shin and HT Kim designed the study. I Heo, M-S Hwang and E-H Hwang developed the search strategy, S-Y Park and B-C Shin wrote the manuscript. All authors critically revised the protocol and approved the final manuscript.

**Conceptualization:** Sun-Young Park, Byung-Cheul Shin.

**Writing – original draft:** Sun-Young Park, Byung-Cheul Shin.

**Writing – review & editing:** Hyun-Tae Kim, In Heo, Man-Suk Hwang, Eui-Hyoung Hwang.
